# Associations Between Coronal Alignment, Patellar Height, Chondrocalcinosis and Radiographic Severity of Knee Osteoarthritis in a Single-Center Cross-Sectional Clinical Cohort

**DOI:** 10.3390/medicina62020396

**Published:** 2026-02-18

**Authors:** Laszlo Irsay, Theodor Popa, Madalina Gabriela Iliescu, Cosmina Ioana Bondor, Alina Deniza Ciubean, Viorela Mihaela Ciortea

**Affiliations:** 1 Department of Physical Medicine, Balneotherapy and Rehabilitation, “Iuliu Hațieganu” University of Medicine and Pharmacy, 400347 Cluj-Napoca, Romania; laszlo.irsay@umfcluj.ro (L.I.); viorela.ciortea@umfcluj.ro (V.M.C.); 2 Department of Rehabilitation Medicine, Clinical Rehabilitation Hospital, 400347 Cluj-Napoca, Romania; popa.theo94@gmail.com; 3 Department of Rehabilitation Medicine, Faculty of Medicine, “Ovidius” University of Constanta, 900470 Constanta, Romania; iliescumadalina@gmail.com; 4 Department of Medical Informatics and Biostatistics, Faculty of Medicine, “Iuliu Hațieganu” University of Medicine and Pharmacy, 400347 Cluj-Napoca, Romania; cosmina_ioana@yahoo.com

**Keywords:** knee osteoarthritis, chondrocalcinosis, patellar height, radiographic severity

## Abstract

*Background and Objectives*: Knee osteoarthritis (OA) is a leading cause of pain and disability, with radiographic severity influenced by age, biomechanical alignment, and structural joint features. Data describing the association between common radiographic parameters and OA severity in Eastern European clinical populations remain limited. This study aimed to evaluate the associations between radiographic OA severity and coronal alignment, patellar height, and chondrocalcinosis in a Romanian clinical cohort. *Materials and Methods*: This single-center cross-sectional study included adult patients undergoing knee radiography for knee-related symptoms and/or functional assessment at a rehabilitation hospital between 2023 and 2025. Radiographs were obtained in the supine, non-weight-bearing position and included anteroposterior and lateral views. OA severity was graded using the Kellgren–Lawrence (KL) classification. Coronal alignment was assessed using the femorotibial angle, patellar height using the Insall–Salvati ratio (ISR), and chondrocalcinosis was recorded as present or absent. Associations between radiographic parameters and KL grade were analyzed using non-parametric statistics. Receiver operating characteristic (ROC) analyses were performed for exploratory assessment of limited separation between distributions. *Results*: Moderate to severe OA (KL ≥ 3) was present in 49% of patients. KL grade showed a moderate positive correlation with age (r = 0.50, *p* < 0.001) and differed significantly across coronal alignment categories (*p* < 0.001). Varus/valgus and pathological alignment classifications demonstrated moderate sensitivity (0.69–0.85) and variable specificity (0.52–0.85) for higher KL grades. ROC analyses of continuous alignment and ISR measures yielded area under the curve values ranging from approximately 0.65 to 0.68, indicating limited separation between distributions. Radiographically detected chondrocalcinosis was present in 5.3% of patients and showed no significant association with OA severity, and neither did patellar height. *Conclusions*: In this single-center Romanian clinical cohort, radiographic OA severity was associated with coronal plane alignment but not with patellar height or chondrocalcinosis. Alignment measures demonstrated limited discriminative ability and should be interpreted as complementary rather than diagnostic indicators of OA severity. These findings provide descriptive radiographic data from an Eastern European clinical population and highlight the need for longitudinal and population-based studies incorporating mechanical axis assessment and functional outcomes.

## 1. Introduction

Knee osteoarthritis (OA) is among the most prevalent musculoskeletal disorders worldwide and a major contributor to pain, disability, and reduced quality of life in older adults. Its burden continues to increase due to population aging and the rising prevalence of obesity and chronic musculoskeletal conditions [1,2]. Radiographic assessment remains central to OA evaluation, particularly in large clinical cohorts, as it allows standardized documentation of structural joint changes.

OA severity on conventional radiographs is commonly graded using the Kellgren–Lawrence (KL) classification, which reflects osteophyte formation, joint space narrowing, and subchondral bone changes [3]. However, radiographic severity does not always correlate with pain intensity or functional impairment, suggesting that structural damage alone does not fully explain the clinical expression of the disease [4]. Recent evidence indicates that biomechanical alignment and functional loading patterns play a critical role in shaping OA progression and rehabilitation outcomes, highlighting the importance of integrating biomechanical parameters into radiographic assessment [3].

Coronal plane malalignment is a well-established factor associated with altered knee joint loading and OA progression. Varus alignment increases medial compartment loading, while valgus alignment preferentially affects the lateral compartment [5,6]. Although the mechanical hip–knee–ankle axis represents the gold standard for alignment assessment, it requires full-length standing radiographs that are not routinely available in many clinical settings. As a result, surrogate measures such as the femorotibial angle on standard knee radiographs are frequently used, despite their known limitations in representing true mechanical alignment [7].

Patellar height, commonly quantified using the Insall–Salvati ratio (ISR), influences patellofemoral biomechanics and has been linked to anterior knee pain and patellofemoral joint degeneration [8,9]. However, its relevance to tibiofemoral OA severity remains uncertain, particularly when evaluated using grading systems primarily focused on the tibiofemoral compartments, such as the KL classification. Similarly, chondrocalcinosis related to calcium pyrophosphate deposition disease often coexists with OA but may become difficult to detect radiographically in advanced degenerative stages, potentially limiting its observed association with OA severity [10,11].

Data describing how these radiographic parameters relate to OA severity in Eastern European clinical populations are limited. Existing Romanian studies have primarily focused on prevalence or radiographic and clinical characteristics of knee osteoarthritis, with fewer analyses integrating multiple radiographic features within the same cohort [12,13]. Moreover, most available data is derived from symptomatic or rehabilitation-based samples, underscoring the need for cautious interpretation of population-level generalizations.

Therefore, the objective of this study was to evaluate the associations between radiographic OA severity and coronal alignment, patellar height, and chondrocalcinosis in a single-center Romanian clinical cohort.

We hypothesized that:

**a.**  *Higher KL grades would be associated with coronal plane malalignment.*

**b.**  *Patellar height would show limited association with tibiofemoral OA severity as assessed by the KL classification.*

**c.**  *Chondrocalcinosis would demonstrate low radiographic prevalence and limited association with advanced OA severity due to reduced detectability in severe disease stages.*

## 2. Materials and Methods

This study was conducted and reported in accordance with the Strengthening the Reporting of Observational Studies in Epidemiology (STROBE) guidelines for cross-sectional studies.

### 2.1. Study Design and Setting

This was a single-center, retrospective, cross-sectional observational study conducted at the Public Rehabilitation Hospital of Cluj-Napoca, Romania. The study evaluated knee radiographs obtained as part of routine clinical care between January 2023 and December 2025. The hospital is a tertiary rehabilitation center that receives referrals for musculoskeletal conditions, including knee osteoarthritis.

Ethical approval was obtained from the institutional ethics committee (protocol code 14273/04.08.2025). Informed consent for the use of anonymized clinical data for research purposes was obtained at hospital admission.

### 2.2. Participants

A total of 900 adult patients (≥18 years) were included. Radiographs were selected from the institutional imaging archive using a convenience sampling approach, based on the alphabetical order of patients’ surnames, with approximately 300 radiographs selected per calendar year. This method was chosen to ensure temporal coverage across the study period but does not represent random sampling.

Eligible patients have undergone knee radiography for knee-related musculoskeletal complaints or functional assessment during rehabilitation admission. Inclusion criteria were: (1) availability of standard anteroposterior and lateral knee radiographs, (2) radiographs of sufficient quality to permit measurement of alignment and patellar height. Exclusion criteria were: (1) prior knee arthroplasty or internal fixation, (2) history of major knee trauma resulting in altered anatomy, (3) radiographs with inadequate positioning, motion artifacts, incomplete visualization of anatomical landmarks, or excessive exposure that precluded accurate measurement. When multiple radiographs were available for the same patient, only the most recent examination was analyzed to avoid duplication.

### 2.3. Radiographic Acquisition Protocol

All knee radiographs were obtained using the standard institutional protocol. Imaging included an anteroposterior and a lateral view of the knee. Radiographs were acquired with patients in the supine, non-weight-bearing position, with the knee in extension for the anteroposterior view and approximately 30° of flexion for the lateral view.

Because radiographs were obtained in the supine position, joint space width and coronal alignment measurements do not reflect true weight-bearing conditions. Full-length standing lower-limb radiographs were not available; therefore, true mechanical axis assessment using the hip–knee–ankle (HKA) angle was not performed. All alignment measurements were performed on supine, non-weight-bearing radiographs and represent anatomical surrogates rather than functional or mechanical alignment. These measurements do not capture load-dependent alignment behavior and were therefore not intended to reflect in vivo biomechanical conditions.

### 2.4. Radiographic Evaluation and Measurements

Radiographic images were reviewed using DICOM Viewer version 1.0.0.107. All measurements were performed by a single physician with formal training in musculoskeletal radiology. The evaluator had access only to the radiographic images and was not provided with additional clinical information beyond patient age and sex.

OA severity was graded using the Kellgren–Lawrence (KL) classification, ranging from grade 0 (no radiographic OA) to grade 4 (severe OA), based on the presence of osteophytes, joint space narrowing, subchondral sclerosis, and bone deformity [3].

**Figure 1:**
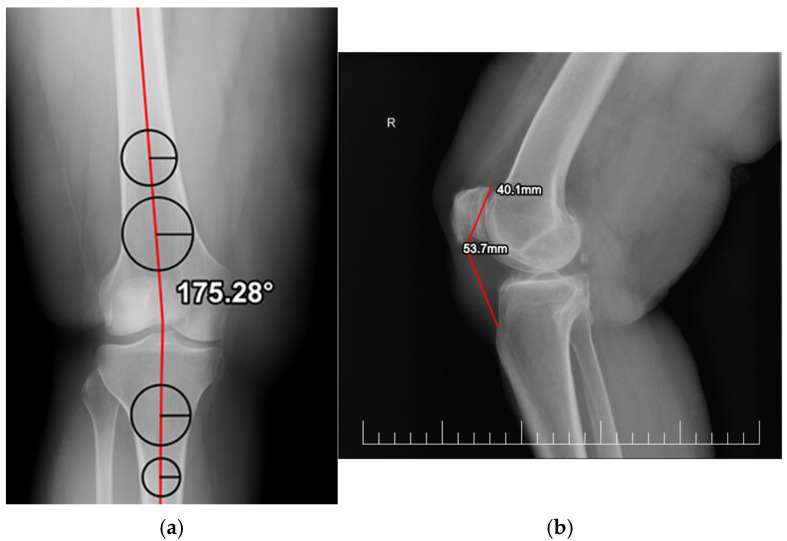
(**a**). Knee alignment measurement; (**b**). Patellar height measurement.

Coronal alignment was assessed using the femorotibial angle, measured on anteroposterior knee radiographs following the method described by Zampogna et al. [7]. Two circles were drawn at predefined distances from the joint line along the femoral and tibial diaphyses, and mid-diaphyseal lines were constructed by connecting their centers. The angle formed by the intersection of these lines was recorded in degrees (Figure 1a). This measurement represents an anatomical alignment surrogate and does not correspond to the true mechanical axis of the lower limb. Alignment was categorized as varus or valgus based on the direction of deviation and further classified as normal or pathological using predefined angular thresholds derived from published literature [7]. These categories were used for exploratory association analyses only.

Patellar height was evaluated on lateral radiographs using the Insall–Salvati ratio (ISR), calculated as the ratio between patellar tendon length and patellar length [14]. Measurements were performed with the knee flexed at approximately 30°. ISR values were categorized as follows: <0.8: patella baja, 0.8–1.2: normal patellar height, >1.2: patella alta (Figure 1b).

Chondrocalcinosis was assessed as a binary variable (present/absent) based on the identification of linear or punctate calcifications within the hyaline or fibrocartilage on standard radiographs. No grading system was applied. “Metabolic disease” was defined as the presence of radiographically visible calcium pyrophosphate deposition consistent with chondrocalcinosis. The authors acknowledge that detection of chondrocalcinosis is limited on standard radiographs, particularly in advanced OA stages where degenerative changes may obscure calcifications.

### 2.5. Statistical Analysis

The characteristics of the groups were described using the median and interquartile range for continuous, non-normally distributed data, and absolute and relative frequencies for qualitative data, including ordinal variables. The Spearman correlation coefficient was used to assess the relationship between ordinal variables and continuous non-normally distributed variables, and these associations were illustrated with scatter charts. The diagnostic performance of binary variables was assessed through sensitivity and specificity, along with their 95% confidence intervals (CI). For exploratory purposes, multiple cut-off points were examined to descriptively compare radiographic parameters across OA severity categories; these analyses were not predefined as diagnostic or prognostic evaluations. Using the disease prevalence estimated from the sample, the positive predictive value (PPV) and negative predictive value (NPV) were also calculated. As the PPV and NPV depend on disease prevalence, the authors assume that the study sample is representative of individuals seeking radiological evaluation for symptoms of arthrosis; therefore, if prevalence differs in another population, the reported PPVs and NPVs should not be applied.

The diagnostic performance of continuous variables was evaluated using Receiver Operating Characteristic (ROC) curves, and the area under the curve (AUC) with its 95% CI was reported. Sensitivity and specificity corresponding to the identified cut-off values were also presented. A *p*-value < 0.05 was considered statistically significant. The primary outcome was radiographic OA severity assessed by the Kellgren–Lawrence grade, with coronal alignment as the primary explanatory variable. All ROC analyses and threshold-based classifications were performed for exploratory purposes only. SPSS 25.0 and Excel 2016 were used for data analysis. ROC curves were used solely as descriptive tools to visualize overlap between radiographic parameter distributions across KL categories. The 2-way Contingency Table Analysis online resource was used to calculate the 95% CI for the diagnostic test parameters. All sensitivity, specificity, PPV, and NPV values are reported for descriptive purposes only and should not be interpreted as evidence of diagnostic or prognostic utility.

### 2.6. Study Flow

**Figure 2:**
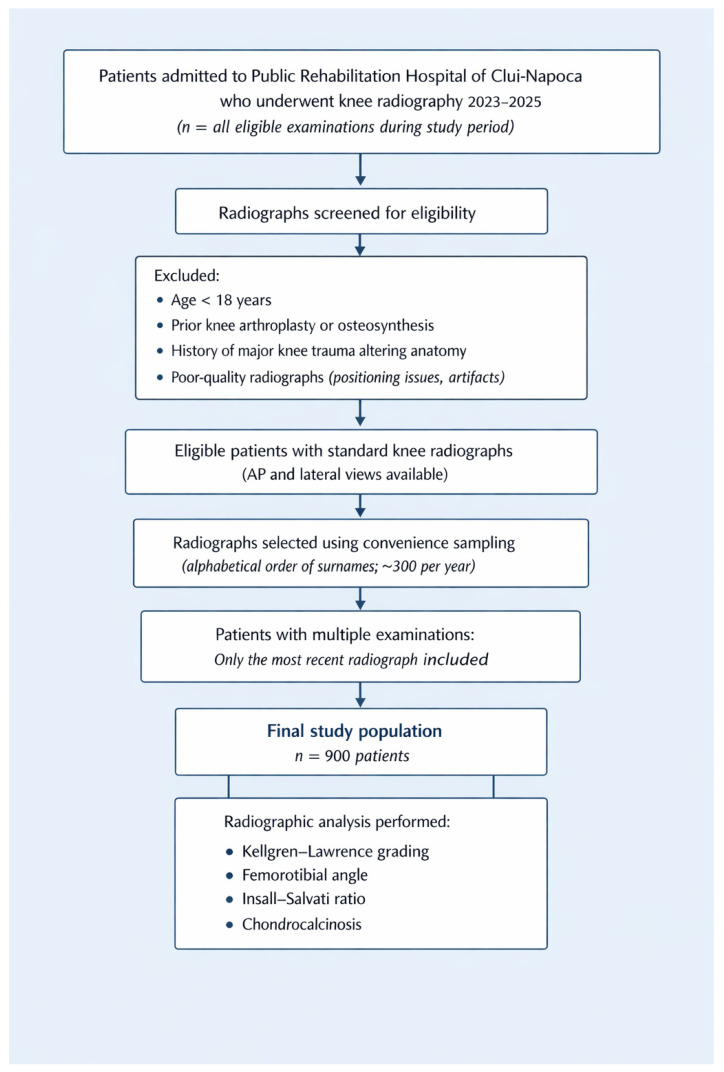
Study flow diagram.

A study flow diagram summarizing patient selection, exclusions, and final inclusion is provided in Figure 2.

## 3. Results

The study included 900 patients who met the inclusion criteria. The main demographic and radiographic characteristics of the cohort are summarized in Table 1. The median age of the study population was 68 years ([IQR]: 59–75). Radiographic OA severity according to the Kellgren–Lawrence (KL) classification showed that 49% of patients had moderate to severe OA (KL ≥ 3). Patella alta was observed in a substantial proportion of patients, whereas patella baja was rare. Chondrocalcinosis (“metabolic disease”) was identified in a small subset of the cohort.

### 3.1. Kellgren–Lawrence Score

No statistically significant differences in Kellgren–Lawrence (KL) scores were observed between sexes (*p* = 0.660). Age showed a moderate positive correlation with KL score (r = 0.50, *p* < 0.001). Neither the presence of radiographically detected metabolic disease (*p* = 0.175) nor patellar position (alta or baja; *p* = 0.166) was associated with KL grade. In addition, KL grade did not differ according to the year of radiographic evaluation (*p* = 0.340).

**Figure 3:**
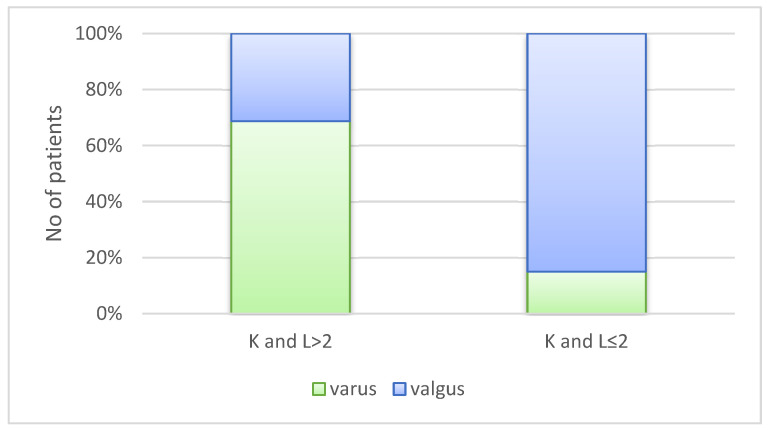
Kellgren and Lawrence score and varus/valgus classification.

An association was observed between KL grade and coronal alignment category (varus vs. valgus) (*p* < 0.001). KL grade was used as a reference to descriptively examine the distribution of alignment patterns across OA severity levels. Among the 459 patients with KL scores ≤ 2, 390 exhibited valgus alignment, whereas 101 of the 441 patients with KL scores > 2 presented with varus alignment. When these categories were compared for exploratory descriptive purposes, 303 patients with KL scores > 2 were classified as having varus alignment, while 138 patients with KL scores ≤ 2 also showed varus alignment, and 69 patients with KL scores > 2 exhibited valgus alignment (Figure 3).

Sensitivity, specificity, positive predictive value (PPV), and negative predictive value (NPV) are presented in Table 2. In our cohort, the prevalence of KL > 3 was 49%. Although the varus/valgus test demonstrated low sensitivity for identifying KL grades 3–4, specificity was acceptable. As expected, PPV and NPV were influenced by the underlying prevalence, in this case reflecting a population referred to specialist services for osteoarthritis evaluation.

### 3.2. Varus/Valgus Alignment

**Figure 4:**
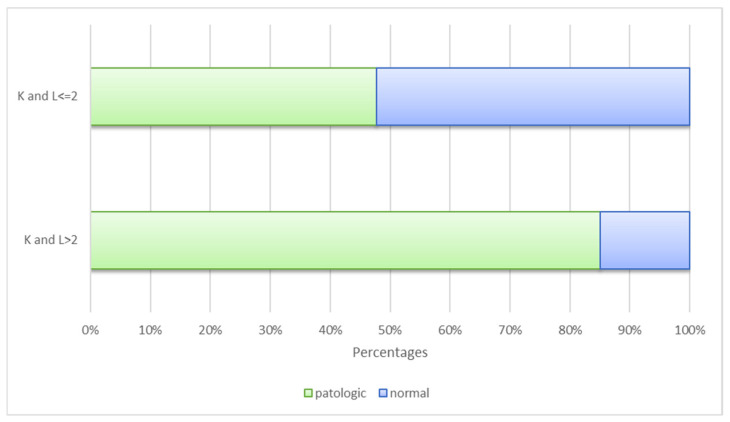
Kellgren and Lawrence score and varus/valgus classification.

When varus/valgus alignment was classified as either pathological or normal, this classification demonstrated a significant association with the KL score (*p* < 0.001). For exploratory comparison, KL grade 2 was used as a reference threshold to examine differences between normal and pathological alignment categories. Among the 159 patients with KL scores ≤ 2, 240 were evaluated as having normal alignment. Conversely, of the 441 patients with KL scores > 2, 375 were classified as having pathological alignment. When distributions across KL categories were examined, a higher proportion of patients with KL > 2 were classified as having varus alignment. (Figure 4). Sensitivity, specificity, positive predictive value (PPV), and negative predictive value (NPV) for this exploratory classification are presented in Table 3. In this model, sensitivity for detecting KL grades 3–4 was acceptable, whereas specificity was poor.

### 3.3. Frontal Angle

**Figure 5:**
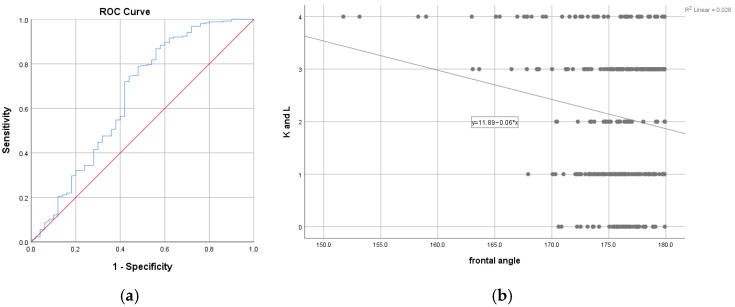
Frontal angle and Kellgren and Lawrence score. (**a**). ROC curve for the association between Kellgren and Lawrence score and frontal angle. (**b**). Correlation between frontal angle and Kellgren and Lawrence score.

When examined in relation to frontal angle, more advanced radiographic osteoarthritis (KL > 3) was more frequently observed among patients with lower frontal angle values. In exploratory receiver operating characteristic (ROC) analysis, a frontal angle threshold of 173.17° yielded an area under the curve (AUC) of 0.65 (95% CI: 0.56–0.75, *p* = 0.001), reflecting limited separation between distributions (Figure 5a). Among the 750 patients with KL scores ≤ 3, 651 exhibited a frontal angle > 173.17°, whereas 66 of the 150 patients with KL scores > 3 had a frontal angle ≤ 173.17°. Using this threshold for descriptive comparison, 66 patients with KL grade 4 were observed below the threshold, 84 patients with KL ≤ 3 also fell below this value, and 99 patients with KL grade 4 were above the threshold. Sensitivity, specificity, positive predictive value (PPV), and negative predictive value (NPV) associated with this threshold are presented in Table 4 and should be interpreted as exploratory. Sensitivity for identifying KL grade 4 was low, while specificity was modest. As illustrated in Figure 5b, no significant linear correlation was observed between KL score and frontal angle (Spearman r = −0.02, *p* = 0.71).

### 3.4. ISR

**Figure 6:**
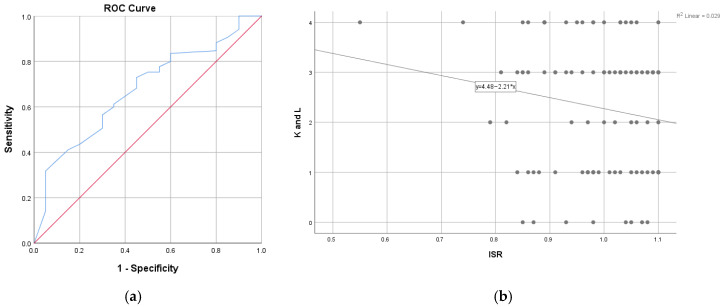
ISR and Kellgren and Lawrence score. (**a**). ROC curve for the association between Kellgren and Lawrence score with 0, 1, 2 or 3 as reference value versus 4 and ISR in group with ISR ≤ 1.1. (**b**). Correlation between ISR and Kellgren and Lawrence score in group with ISR ≤ 1.1.

When analyses were restricted to patients with an Insall–Salvati ratio (ISR) ≤ 1.1, higher radiographic severity (KL > 3) was more frequently observed among those with lower ISR values. In exploratory ROC analysis, an ISR threshold of 0.97 yielded an area under the curve (AUC) of 0.68 (95% CI: 0.55–0.80, *p* = 0.015), indicating limited separation between distributions (Figure 6a). Among the 250 patients with KL scores ≤ 3, 174 had an ISR > 0.97, whereas 33 of the 60 patients with KL scores > 3 exhibited an ISR ≤ 0.97. Using this threshold for descriptive comparison, 33 patients with KL grade 4 were observed below the threshold, 27 patients with KL ≤ 3 also fell below this value, and 81 patients with KL grade 4 were observed above the threshold.

Sensitivity, specificity, positive predictive value (PPV), and negative predictive value (NPV) corresponding to this threshold are summarized in Table 5 and should be interpreted as exploratory. Sensitivity for identifying KL grade 4 was low, and specificity was also limited. As shown in Figure 6b, no significant linear correlation was observed between KL score and ISR within the evaluated subgroup (ISR ≤ 1.1) (Spearman r = −0.16, *p* = 0.103).

## 4. Discussion

The present study provides a comprehensive radiographic description of knee osteoarthritis (OA) in a Romanian clinical cohort by integrating measures of structural severity (Kellgren–Lawrence classification), coronal plane alignment, patellar height, and radiographic evidence of chondrocalcinosis. These findings add to the existing literature by describing how commonly used radiographic parameters relate to OA severity within a single-center Eastern European clinical setting. To our knowledge, this is one of the few studies in Eastern Europe—and the first in Romania—to simultaneously evaluate structural OA severity, coronal alignment, patellar height, and chondrocalcinosis within the same radiographic dataset. By examining multiple radiographic features in parallel, this study offers a more detailed descriptive assessment than the single-parameter approaches commonly reported in previous Romanian studies.

We found that nearly half of all evaluated knees (49%) demonstrated a Kellgren–Lawrence (KL) score ≥ 3, indicating moderate to severe OA. This prevalence is consistent with the upper range of Romanian clinic-based studies, such as Dumitru et al., who reported KL ≥ 3 in 40–55% of symptomatic patients, depending on age and comorbidity profiles [12]. Compared with large international cohorts, such as the Framingham Osteoarthritis Study and the Rotterdam Study, where KL ≥ 3 prevalence in adults over 60 ranged from 19% to 34%, Romanian patients appear to present with more advanced radiographic disease at the time of evaluation [6,15]. These differences may reflect variations in healthcare access, referral patterns, and lifestyle or occupational factors, including high physical workload among older Romanian adults.

It is important to acknowledge that the study population was drawn from a rehabilitation-based clinical setting rather than from the general community. As a result, the relatively high proportion of advanced radiographic disease likely reflects the characteristics of patients seeking care for symptomatic or function-limiting knee conditions. This pattern is consistent with previous reports showing that clinic-based cohorts tend to exhibit higher Kellgren–Lawrence grades than population-based samples. While this limits the generalizability of the findings beyond similar clinical settings, it provides relevant insight into the structural disease burden among patients referred for rehabilitation within the Romanian healthcare system [2,13,16].

Age demonstrated a moderate correlation with Kellgren–Lawrence (KL) score (r = 0.50), consistent with previous reports identifying age as a major determinant of radiographic osteoarthritis (OA) severity and progression [1,16]. No significant sex-related differences in KL distribution were observed in this cohort, contrasting with population-based studies in which women often exhibit higher OA prevalence and severity. This discrepancy may reflect characteristics of the rehabilitation-based clinical sample rather than biological differences.

Radiographically detected chondrocalcinosis did not show a significant association with KL grade. Although calcium pyrophosphate deposition disease (CPPD) frequently coexists with OA, prior studies have demonstrated that chondrocalcinosis becomes increasingly difficult to detect on standard radiographs in advanced disease stages, where osteophytes and subchondral sclerosis may obscure calcifications [11]. The relatively low prevalence and lack of association observed in this cohort are therefore likely influenced by limitations of radiographic detection rather than absence of metabolic involvement. More sensitive imaging modalities such as ultrasound or dual-energy computed tomography may be required to clarify this relationship [10,11,15].

Coronal plane alignment abnormalities were significantly associated with higher KL grades, supporting existing evidence that alignment deviations commonly accompany more advanced structural disease [5,6]. However, alignment assessment was based on the femorotibial angle measured on supine radiographs, which represent an anatomical surrogate rather than the true mechanical axis. Exploratory analyses showed that alignment classifications exhibited moderate sensitivity and variable specificity for higher KL grades, while ROC analyses yielded modest AUC values (~0.65), indicating limited separation between distributions. These findings are consistent with previous studies showing that static alignment measures alone do not reliably distinguish OA severity at the individual level and should be interpreted as supportive contextual information rather than diagnostic indicators [7,17,18].

Patellar height, assessed using the Insall–Salvati ratio (ISR), was not associated with tibiofemoral OA severity across the cohort. Although patella alta was frequently observed, this lack of association likely reflects the limited sensitivity of the KL classification to patellofemoral joint pathology. Previous studies linking patellar height to OA have primarily focused on patellofemoral disease or anterior knee pain populations rather than mixed-compartment radiographic OA [8,9]. Consequently, the clinical relevance of patellar height may be underestimated when tibiofemoral-focused grading systems are used.

The absence of a relationship between ISR and KL grade does not exclude clinical relevance of patellar height. Patellar positioning primarily affects patellofemoral biomechanics, instability, and anterior knee symptoms—domains not directly evaluated in this study and not adequately captured by the KL system [8,9,14,19]. Future studies incorporating patellofemoral-specific imaging or magnetic resonance imaging may better clarify the role of patellar height in compartment-specific disease patterns.

Overall, the findings indicate that coronal alignment abnormalities are more frequently observed in patients with advanced radiographic OA, while patellar height and chondrocalcinosis show limited association with tibiofemoral structural severity when assessed using standard radiographs. The modest limited separation between distributions of alignment and patellar height measures underscores that these parameters should be considered complementary descriptors rather than stand-alone indicators of disease stage.

Emerging research suggests that dynamic biomechanical factors and advanced imaging modalities may provide additional insight into OA progression beyond static radiographic measures [18]. Incorporating weight-bearing mechanical axis assessment, gait analysis, and MRI-based evaluation in future longitudinal studies may help clarify the complex relationships between alignment, structural damage, and clinical outcomes, particularly in rehabilitation-based clinical populations.

Several limitations should be noted. First, the cross-sectional design precludes any inference regarding causality or temporal relationships between alignment abnormalities and OA progression. Second, all radiographs were obtained in the supine, non-weight-bearing position, limiting the validity of joint space and alignment assessment compared with weight-bearing or full-length mechanical axis radiographs. Coronal alignment was evaluated using the femorotibial angle, which represents anatomical alignment and does not reflect the true mechanical axis of the lower limb. Third, the study used a single-center convenience sample drawn from a rehabilitation hospital, introducing selection bias and limiting generalizability beyond similar clinical settings. The alphabetical selection method does not constitute random sampling. Fourth, multiple statistical comparisons were performed without formal correction, and all analyses should therefore be interpreted as exploratory. ROC analyses were applied to radiographic parameters measured simultaneously with KL grading and do not establish diagnostic accuracy. Fifth, radiographic assessment was performed by a single evaluator without formal intra- or inter-rater reliability analysis, which may introduce measurement bias. Sixth, chondrocalcinosis assessment was limited to standard radiographs and may underestimate true prevalence, particularly in advanced OA stages. Seventh, no formal adjustment for multiple comparisons was applied, and all statistical findings should therefore be interpreted as exploratory and hypothesis-generating. Finally, potential confounding factors such as body mass index, occupational exposure, and functional status were not available and could not be adjusted for in the analyses.

## 5. Conclusions

In this single-center cross-sectional clinical cohort, higher radiographic severity of knee osteoarthritis was associated with coronal plane alignment abnormalities but not with patellar height or chondrocalcinosis. Alignment measures demonstrated limited separation between distributions and should be considered supportive descriptive parameters rather than diagnostic indicators of disease severity.

The findings do not establish causal relationships, do not define radiographic phenotypes, and cannot be generalized to the broader Romanian population. Beyond reporting associations, these findings provide a structured radiographic description of a rehabilitation-based clinical cohort and may inform the design of future longitudinal studies integrating weight-bearing alignment and functional outcomes. These findings should not be extrapolated beyond similar rehabilitation-based clinical settings.

## Figures and Tables

**Table 1:** Characteristics of the study group.

Parameters	Total Group (*n* = 900)
Male, no. (%)	306 (11.3)
Age	68 (59; 75)
Kellgren and Lawrence score	
0, no. (%)	111 (12.3)
1, no. (%)	261 (29)
2, no. (%)	87 (9.7)
3, no. (%)	291 (32.3)
4, no. (%)	150 (16.7)
Patellar position	
alta, no. (%)	366 (40.7)
baja, no. (%)	9 (1)
normal, no. (%)	525 (58.3)
Insall Salvati Ratio	1.17 (1.06; 1.30)
Frontal angle	176.26 (173.88, 178.14)
Varus, no. (%)	372 (41.3)
Varus/valgus pathologic, no. (%)	594 (66)
Metabolic disease, no. (%)	47.7 (5.3)

**Table 2:** Parameters of the exploratory classification varus/valgus for Kellgren and Lawrence scores higher than 2 with their 95% confidence intervals and the prevalence of the Kellgren and Lawrence scores higher than 2 estimated from the sample.

Statistics	Value (95% CI)
Sensitivity	0.69 (0.63; 0.73)
Specificity	0.85 (0.80; 0.89)
Positive Predictive Value (PPV)	0.82 (0.75; 0.87)
Negative Predictive Value (NPV)	0.74 (0.69; 0.78)
Prevalence (estimated from sample)	0.49 (0.43; 0.55)

**Table 3:** Parameters of the exploratory classification varus/valgus for Kellgren and Lawrence scores higher than 2 with their 95% confidence intervals.

Statistics	Value (95% CI)
Sensitivity	0.85 (0.80; 0.90)
Specificity	0.52 (0.47; 0.57)
Positive Predictive Value (PPV)	0.63 (0.59; 0.67)
Negative Predictive Value (NPV)	0.78 (0.71; 0.85)

**Table 4:** Parameters of the exploratory classification for the frontal angle cut-off ≤ 173.17 for Kellgren and Lawrence scores higher than 3, with their 95% confidence intervals.

Statistics for ≤ 173.17 Cut-Off for Frontal Angle	Value (95% CI)
Sensitivity	0.44 (0.32; 0.56)
Specificity	0.87 (0.84; 0.89)
Positive Predictive Value (PPV)	0.17 (0.13; 0.21)
Negative Predictive Value (NPV)	0.89 (0.86; 0.91)
Prevalence of K and L = 4 (estimated from the sample)	0.4 (0.29; 0.51)

**Table 5:** Parameters of the exploratory classification for the ISR cut-off ≤ 0.97 for Kellgren and Lawrence scores higher than 3 with their 95% confidence intervals in the group with ISR ≤ 1.1.

Statistics for 0.97 Cut-Off for ISR	Value (95% CI)
Sensitivity	0.55 (0.34; 0.75)
Specificity	0.68 (0.63; 0.73)
Positive Predictive Value (PPV)	0.29 (0.18; 0.39)
Negative Predictive Value (NPV)	0.87 (0.80; 0.93)
Prevalence (estimated from sample)	0.19 (0.13; 0.28)

## Data Availability

The original contributions presented in this study are included in the article. Further inquiries can be directed to the corresponding author.

## Abbreviations

The following abbreviations are used in this manuscript:

OA	Osteoarthritis
KL	Kellgren–Lawrence
ISR	Insall–Salvati Ratio
CPPD	Calcium Pyrophosphate Deposition Disease
ROC	Receiver Operating Characteristic
AUC	Area Under the Curve
PPV	Positive Predictive Value
NPV	Negative Predictive Value
CI	Confidence Interval
